# Variations in the Gut Microbiota of Stray and Domestic Cats

**DOI:** 10.3390/ani16050724

**Published:** 2026-02-26

**Authors:** Yanan Wu, Chunliu Zhao, Xiran Guo, Jingzhe Cheng, Yiyu Liang, Xiaorui Tang, Zhenyu Peng, Kang Lü, Jiamu Ding, Xiaojuan Xu

**Affiliations:** 1 School of Biology and Food Engineering, Hefei Normal University, Hefei 230601, China; x24201015@stu.ahu.edu.cn (Y.W.); 13131700128@163.com (C.Z.); guo_xr03@163.com (X.G.); 13343075650@163.com (J.C.); 13013081303@163.com (Z.P.);; 2 School of Ecology and Environment, Anhui University, Hefei 230601, China

**Keywords:** stray cats, domestic cats, gut microbiota, pathogenic microorganisms

## Abstract

Urban stray cats are in close contact with humans and are important potential vectors for zoonotic diseases. However, comparative studies in the gut microbiota of cats living in different environments remain limited. Here, we conducted a comparative analysis of the gut microbiota between stray and domestic cats using 16S rRNA gene sequencing of fecal samples from 14 stray and 11 domestic cats in Hefei, China. Domestic cats harbored significantly higher alpha diversity (Sobs index, *Padj.* = 0.039; Shannon index, *Padj.* = 0.024) and more complex microbial co-occurrence networks than stray cats. Beta diversity analysis confirmed distinct community structures between the groups. Linear discriminant analysis identified 12 taxa enriched in stray cats, including *Escherichia-Shigella* and other potential opportunistic pathogens. Functional prediction indicated that the gut microbiota of domestic cats was enriched in genes related to DNA repair and cellular structure maintenance, whereas that of stray cats showed higher abundance of functions associated with secondary metabolism, defense mechanisms, and stress response. Phenotypic prediction further revealed increased proportions of stress-tolerant and facultatively anaerobic bacteria in stray cats. These findings demonstrate that lifestyle and environmental exposure shape the feline gut microbiota, with stray cats exhibiting a less diverse, more stress-adapted, and potentially pathogen-enriched microbial profile. This study provides insights into the ecological adaptation of gut microbiota and highlights implications for zoonotic risk and One-Health-oriented management of urban cat populations.

## Simple Summary

Cats living outdoors as strays face very different conditions from those kept as pets—including unpredictable food sources, exposure to pathogens, and lack of veterinary care. This study asked whether these differences affect the bacteria living in the cat’s gut. We compared the gut microbiota of stray and domestic cats using genetic sequencing of fecal samples. We found that domestic cats have more diverse and stable gut bacterial communities. Stray cats, on the other hand, carry more bacteria that can tolerate stress and survive with or without oxygen. Some bacteria linked to potential infections, such as *Escherichia-Shigella*, were also more common in stray cats. The gut bacteria of stray cats appear to be geared toward surviving in harsh environments, while those in domestic cats seem more involved in maintaining general health. These results help us understand how the environment shapes gut health in cats and may guide strategies to reduce disease risks to cats and humans under the “One Health” framework.

## 1. Introduction

Cats (*Felis catus*) are the world’s second most populous companion animals, after domestic dogs (*Canis lupus familiaris*), with a global population estimated to exceed 600 million individuals. Notably, approximately half of them live outdoors either freely or semi-ferally [1]. Although cats are highly beloved for providing companionship, the presence of a large number of stray cats has raised a series of concerns regarding ecology and public health. Stray cats may prey on native wildlife, disrupt ecosystem balance, and carry zoonotic pathogens that can be transmitted to humans and other animals [2,3]. Unlike domestic cats, stray cats usually lack stable sources of nutrition, veterinary care, and hygiene management. They rely on unpredictable food sources and live in environments dense with pathogenic microorganisms, which are potentially important influencing factors for the composition and function of their gut microbiota [4].

The gut microbiota plays an important role in host physiology and is closely associated with nutrient metabolism, immune regulation, maintenance of barrier integrity, and protection against pathogenic invasion [5,6,7,8]. When the gut microbial community becomes dysbiotic, it often results in reduced diversity, decreased ecological stability, and the proliferation of opportunistic pathogens, which together increase the host’s risk of infection, inflammation, or metabolic disorders [9,10]. Previous studies on the gut microbes of cats have mainly focused on potential pathogenic microorganisms. For example, several studies on the gut microbiota of stray cats have confirmed the presence of pathogens, such as *Toxoplasma gondii* and *Salmonella,* in feces, which pose a zoonotic transmission risk and present potential threats to both host health and public health safety [11,12]. Given the important role of the gut microbiota in the host’s health, it is necessary to conduct systematic research on the gut microbiota of cats living in different environments, which is beneficial for their health maintenance and welfare.

So far, only a few studies have been conducted on the gut microbiota of domestic cats. Current research on the gut microbiota of cats mainly focuses on the effects of factors such as diseases, diet, and obesity, and mostly concentrates on domestic cats, with a lack of broad studies on stray cats or cat populations in different environments [13,14,15]. Previous studies have shown that the “core” gut microbiota of domestic cats is mainly composed of Bacteroidota, Firmicutes, and Proteobacteria [16,17]. However, systematic comparative analyses of the gut microbiota between stray and domestic cats remain limited, particularly in terms of the differences in community composition, structure, and functions.

In order to fill the gaps of current studies in cat microbiota, we collected the feces of both domestic and stray cats. We employed 16S rRNA high-throughput sequencing to compare the gut microbiota of the cats living in two different environments. Compared to domestic cats, stray cats usually lack stable sources of nutrition, veterinary care, and hygiene management.Therefore, we hypothesized that (i) there are significant differences in diversity and composition of gut microbiota between stray and domestic cats; (ii) the microbial networks would be simpler and more modular in stray cats; and (iii) the gut of stray cats contains more potential pathogenic microorganisms compared to domestic cats. The findings may provide a scientific basis for advancing research on urban ecological safety and public health within the framework of the “One Health” concept.

## 2. Materials and Methods

### 2.1. Animals and Sampling Sites

A total of 25 cats from Hefei, China, were included in this study, comprising 14 strays and 11 domestic individuals. Stray cat fecal samples were collected from locations with concentrated free-roaming populations, such as university campuses, animal rescue centers, and residential communities, where cats typically lack consistent medical care and stable nutrition. In contrast, domestic cat samples were collected from client-owned pets following their admission to boarding facilities at local pet shops to standardize sampling and minimize pre-analytical variability.

All cats appeared healthy at the time of sampling, with no observable symptoms of gastrointestinal illness. The relevant institutional ethics committee approved the sample collection and experimental protocols.

### 2.2. Sample Collection and Preservation

Fresh fecal samples were collected within half an hour after defecation. All subsequent handling was performed while wearing sterile gloves. Using sterile spatulas, only the interior portion of the feces was collected to avoid the outer material that had been in direct contact with the soil or ground surface. This procedure was designed to minimize environmental contamination and to ensure that the analyzed microbiota accurately represented the host’s intestinal tract. About 5–10 g of feces was placed into sterile tubes already containing absolute ethanol—a step taken to minimize microbial degradation [18].

All samples were then moved to the laboratory under temperature-controlled conditions and finally stored at −80 °C for subsequent analysis [19,20]. We documented relevant details at the time of collection, such as sampling date and sampling location, as shown in Table 1.

### 2.3. DNA Extraction

Total microbial DNA was extracted from each fecal sample using a QIAamp^®^ Fast DNA Stool Mini kit (Qiagen, Hilden, Germany) following the manufacturer‘s protocol. DNA quality and concentration were assessed by agarose gel electrophoresis before sequencing [21,22].

### 2.4. PCR Amplification and Sequencing

The V3-V4 hypervariable region of the bacterial 16S rRNA gene was amplified using the primer pair 338F (5′-ACTCCTACGGGAGGCAGCA-3′) and 806R (5′-GGACTACHVGGGTWTCTAAT-3′), widely used in gut microbiota studies [23,24]. The PCR reaction was carried out in a 25 μL system containing high-fidelity DNA polymerase, buffer, dNTPs, primers, and 1 μL of DNA template. Cycling conditions were as follows: initial denaturation at 98 °C for 2 min, 27 cycles of denaturation at 98 °C for 15 s, annealing at 55 °C for 30 s, extension at 72 °C for 30 s, and a final extension at 72 °C for 5 min. PCR products were purified with a Min Elute PCR Purification kit (Qiagen, Hilden, Germany) and then quantified using the QuantiFluor-ST and the dsDNA System (Promega, Madison, WI, USA). Purified amplicons were pooled in equal amounts, and pair-end 2 × 300 bp sequencing was performed on the Illumina MiSeq PE250 platform (Illumina, San Diego, CA, USA) at Shanghai Majorbio Bio-pharm Technology Co., Ltd. (Shanghai, China) [25,26]. The raw reads were deposited into the NCBI Sequence Read Archive (SRA) database (accession number PRJNA1356635).

### 2.5. Bioinformatics and Statistical Analysis

Raw FASTQ reads were first quality-filtered using fastp (v0.19.6) with a sliding window of 50 bp, trimming low-quality bases (Q < 20). Paired-end reads were merged using FLASH (v1.2.7). Chimeric sequences were removed, and amplicon sequence variants (ASVs) were identified using the DADA2 plugin in QIIME2 (v2023.9.1) [27]. Taxonomic classification was performed using a Naive Bayes Classifier trained on the SILVA database (v138.1) [28].

Alpha diversity indices (Shannon index, ASV richness) and beta diversity metrics (Bray–Curtis and unweighted UniFrac distances) were calculated in QIIME2 (v2023.9.1) [28]. The PICRUSt2 (Phylogenetic Investigation of Communities by Reconstruction of Unobserved States 2, v2.5.2) was used to predict the COG (Clusters of Orthologous Groups) functions of the microbial community [29]. In addition, BugBase phenotype prediction was used to evaluate wide-scale phenotypic properties of the gut microbiota [30].

The Wilcoxon rank-sum test was used to evaluate the differences in the alpha diversity index and phenotypic prediction results between the two study groups. The differences of predicted reads in the COG classifications between the two study groups were evaluated by Welch’s *t*-test. Differences were considered statistically significant at *p* < 0.05 after Benjamini–Hochberg false discovery rate (FDR) correction [31]. Principal coordinate analysis (PCoA) and permutational multivariate analysis of variance (PERMANOVA) were used to evaluate beta diversity [8,32]. The phylum, class, order, family, and genus of the gut microbiota enriched in each study group were identified using linear discriminant analysis effect size (LEfSe) [25], with a threshold set at an LDA score > 2 and *p* < 0.05. Co-occurrence networks were constructed based on Spearman correlation coefficients (FDR-corrected, psych package v2.3.9) and visualized using Gephi 0.10.1. All statistical analyses were conducted in R (v4.3.3) using the vegan (v2.6-4), made4 (v1.78.0), and microeco packages (v1.8.0). For data visualization, including boxplots, principal coordinate analysis, and Venn diagrams, the ggplot2 package (v3.4.4) was used.

## 3. Results

### 3.1. Analysis of 16S rRNA Gene Sequencing Data

**Figure 1:**
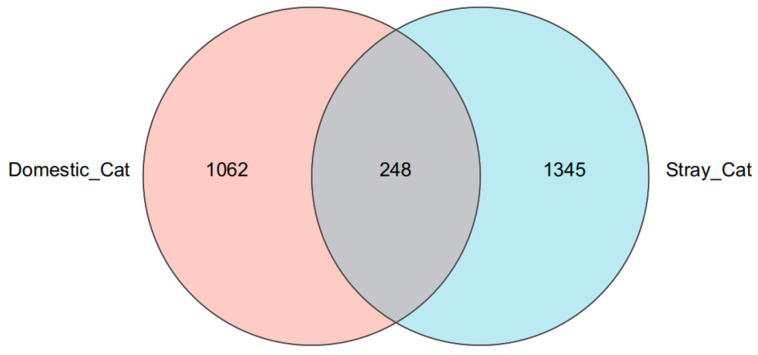
Venn diagram of ASV distribution of bacteria of stray cats and domestic cats.

A total of 600,850 quality filtered sequences were obtained after the detection of 25 samples (Table 2). The average number of sequences for each sample is 24,034. After clustering at 97% similarity, the sequencing data from both stray and domestic cats yielded a total of 2655 amplicon sequence variants (ASVs) (Figure 1). Among these, stray cats accounted for 1593 ASVs, while domestic cats showed 1310. Only 248 ASVs were shared between the two groups.

### 3.2. Gut Microbiota Composition of Stray and Domestic Cats

**Figure 2:**
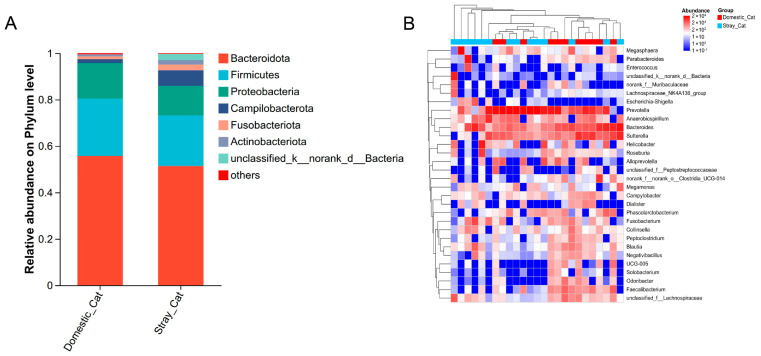
The distributions of gut microbiota composition in stray and domestic cats. Stacked bar chart of relative abundances at the phylum level. Sequences that do not fall into any known groups are labeled as “unassigned”, and groups with an average relative abundance lower than 0.1% are classified as “others” (**A**). Heat map analysis at the genus level, with color intensity representing relative abundance (red indicates high abundance and blue indicates low abundance). Sample types are distinguished by color labels at the top of the heat map (blue for stray cats and orange for domestic cats) (**B**).

Across all fecal samples, we identified a total of 14 bacterial phyla, 57 families, 154 genera, and 2655 amplicon sequence variants (ASVs). Bacteroidota emerged as the most dominant phylum (mean relative abundance ± SD; stray, 51.32% ± 26.54%; domestic, 55.66% ± 20.13%), followed by Firmicutes (stray, 21.87% ± 21.40%; domestic, 24.70% ± 15.87%), Proteobacteria (stray,12.37% ± 13.25%; domestic, 15.34% ± 10.38%), and Campilobacterota (stray, 6.75% ± 12.43%; domestic, 1.69% ± 1.88%) (Figure 2A). The predominant families were Prevotellaceae (stray, 33.65% ± 33.38%; domestic, 39.64% ± 28.44%), Bacteroidaceae (stray, 13.12% ± 15.47%; domestic, 12.15% ± 9.22%), and Veillonellaceae (stray, 5.92% ± 8.74%; domestic, 12.19% ± 9.07%). At the genus level, the samples were dominated by *Prevotella* (stray, 32.37% ± 32.92%; domestic, 34.40% ± 24.90%), *Bacteroides* (stray, 13.12% ± 15.47%; domestic, 12.15% ± 9.22%), and *Sutterella* (stray, 8.71% ± 8.85%; domestic, 12.00% ± 9.24%) (Figure 2B).

### 3.3. Comparative Study of Gut Microbiota in Stray and Domestic Cats

#### 3.3.1. Analysis of Alpha and Beta Diversities

**Figure 3:**
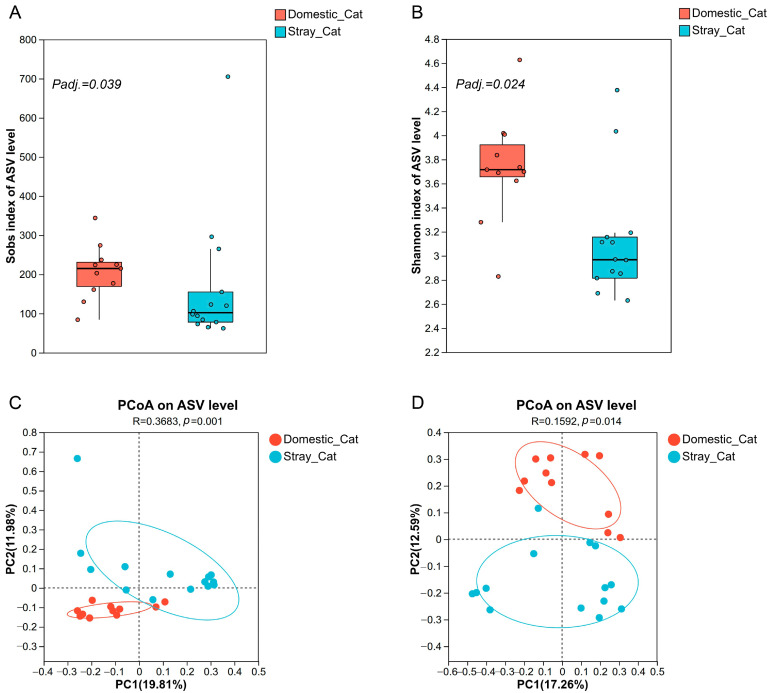
Differences in bacterial diversity between stray and domestic cats. Comparison of amplicon sequence variant (ASV) richness (**A**). Comparison of Shannon diversity index (**B**). Differentiation of bacterial microbiota structure based on Bray–Curtis distances (**C**) and based on unweighted UniFrac distances (**D**). Principal coordinates analysis (PCoA) was used to show patterns across sample groups. Adonis tests were performed on Bray–Curtis and unweighted UniFrac distances, respectively. Significance was set at the 0.05 level.

Our analysis revealed clear differences in alpha diversity between the two groups (Figure 3A,B). Domestic cats showed significantly higher values in both the Sobs index (Wilcoxon rank-sum test, *Padj.* = 0.039) and the Shannon index (Wilcoxon rank-sum test, *Padj.* = 0.024) compared to stray cats, suggesting that owned cats harbor richer and more diverse gut microbial communities.

To examine between-sample diversity, we performed PCoA based on Bray–Curtis and unweighted UniFrac distances. The resulting ordination plots revealed two distinct clusters, visually separating stray and domestic cat gut microbiota (Figure 3C,D). This separation was statistically supported by ANOSIM, which confirmed significant differences in community structure between the two groups (Bray–Curtis: R = 0.3863, *p* = 0.001; unweighted UniFrac: R = 0.1592, *p* = 0.014).

#### 3.3.2. Differences in Gut Microbiota Composition Between Stray and Domestic Cats

**Figure 4:**
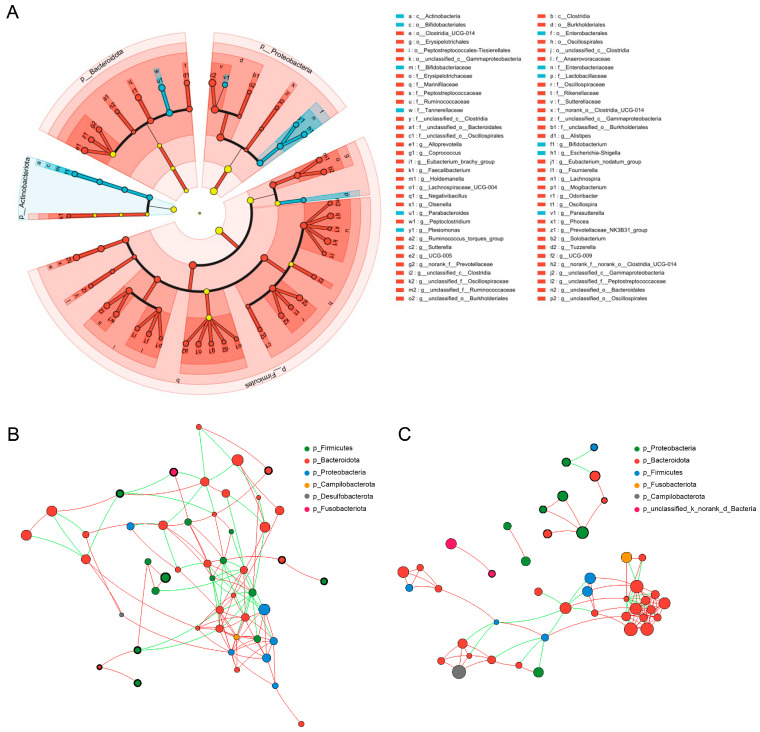
LEfSe results showing significantly enriched taxa in the gut microbiota of domestic and stray cats (**A**), LDA > 2, *p* < 0.05. Co-occurrence networks of domestic (**B**) and stray (**C**) cats, where nodes represent taxa, node size indicates relative abundance, and edges denote correlations, |r| > 0.6 and *p* < 0.05.

Based on linear discriminant analysis effect size (LEfSe) results, we identified 12 bacterial taxa that were significantly enriched in stray cats, compared to 56 in domestic cats, spanning multiple taxonomic ranks from phylum to genus (LDA > 2, *p* < 0.05; Figure 4A). The 12 taxa significantly enriched in the stray cat group included Parabacteroides, Tannerellaceae, Enterobacterales, Enterobacteriaceae, *Escherichia-Shigella*, Actinobacteria, Bifidobacteriaceae, *Bifidobacterium*, Bifidobacteriales, *Plesiomonas*, *Parasutterella*, and Lactobacillaceae. We also constructed bacterial co-occurrence networks for both groups using the top 50 amplicon sequence variants (ASVs), applying a threshold of |r| > 0.6 and *p* < 0.05. The resulting network for domestic cats comprised 258 nodes (Figure 4B), notably more than the 228 nodes observed in stray cats (Figure 4C). This reduction in network complexity suggests that the gut microbial ecosystem in stray cats may be less structurally stable compared to their domestic counterparts.

### 3.4. Variations in Predicted Gut Microbiota Functions Between Stray and Domestic Cats

**Figure 5:**
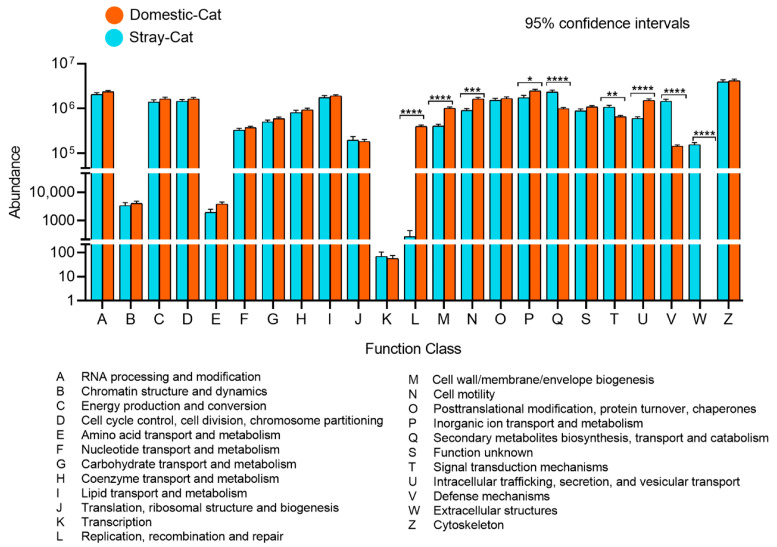
Differences in COG functional annotation between stray and domestic cats. The bar chart shows the extent of differences in COG functional distribution among groups. The values in the bar chart indicate the abundance of functional genes in the metabolic pathway, * *Padj.* < 0.05, ** *Padj.* < 0.01, *** *Padj.* < 0.001, **** *Padj.* < 0.0001.

The results of COG functional prediction revealed significant differences in multiple metabolic and structural functions between the gut microbiota of stray cats and domestic cats (Figure 5). Specifically, functions related to DNA replication, recombination, and repair (L), cell wall/membrane biogenesis (M), cell motility (N), and intracellular trafficking and secretion (U) were significantly enriched in domestic cat samples (Welch’s *t*-test, *p* < 0.05), indicating that their gut microbiota is more inclined toward maintaining cellular structural stability and genetic integrity. In contrast, secondary metabolite synthesis (Q), defense mechanisms (V), signal transduction (T), and extracellular structures (W) were significantly higher in stray cat samples (Welch’s *t*-test, *p* < 0.05), suggesting that their microbiota exhibits stronger defensive and stress-regulatory capabilities in complex environments. These findings indicate that hosts with different lifestyles develop distinct adaptive strategies by shaping the functional profiles of their gut microbiota.

### 3.5. Differences in Phenotypic Properties of the Gut Microbiota Between Stray and Domestic Cats

**Figure 6:**
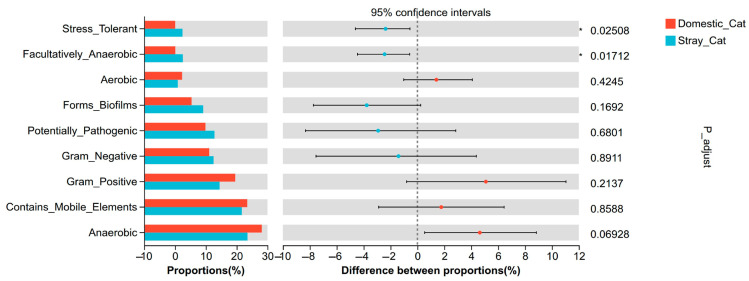
Phenotypic prediction of gut microbiota between stray and domestic cats. * *Padj.* < 0.05.

BugBase phenotype prediction showed that there were nigh phenotypes, including stress-tolerant, facultatively anaerobic, aerobic, forms biofilms, potentially pathogenic, Gram-negative, Gram-positive, contains mobile elements, and anaerobic (Figure 6). Only two phenotypes, stress-tolerant and facultatively anaerobic, have significantly increased in stray cats (*Padj.* < 0.05). Although the overall “Potentially Pathogenic” did not significantly increase in the stray cats, the *Escherichia-Shigella* genus (which includes various opportunistic pathogenic bacteria) was significantly enriched in the gut of stray cats (Figure 4A), suggesting that stray cats may face a greater risk of gut diseases.

## 4. Discussion

The composition, structure, and diversity of gut microbiota are associated with the maintenance of animal health. In this study, we found that both the stray and domestic cats’ gut microbiota were dominated by Bacteroidota, followed by Firmicutes (the total mean relative abundance of these two phyla accounts for more than 70%). This result is consistent with a previous study in domestic and feral cats worldwide [33]. However, it is somewhat different from the gut microbiota detected in the cats living in Malaysia, which were dominated by Firmicutes [34]. At the family and genus levels, Prevotellaceae and its genus *Prevotella*, as well as Bacteroidaceae and its genus *Bacteroides*, were the most abundant in our study samples. This distribution pattern is similar to the result of a study on the healthy fecal “Core Microbiome” of North American pet domestic cats (*Felis catus*) [35]. Nevertheless, in another study on the gut microbiota of stray and pet cats, *Bacteroides* did not rank as the most abundant genus [33]. Our results, together with previous studies, revealed that the main taxonomic groups of the gut microbiota in cats from different regions show certain differences.

Stray cats generally face very different conditions from those kept as pets, such as unpredictable food sources, which might put certain pressure on the stability of their gut microbiota [36]. Our results provide evidence that stray cats exhibit significantly lower microbial diversity and simpler interaction networks compared to domestic cats. This contrasts with previous findings from other studies showing no significant differences in alpha diversity between stray and pet cats [5,34,35]. Given that reduced microbial diversity and structural stability have been demonstrated to be closely associated with gut microbiota imbalance in previous studies [37], our findings indicate that stray cats may be experiencing greater health stress than domestic cats. On the contrary, domestic cats maintain higher species richness and more complex microbial networks, likely due to their stable living conditions, including regular feeding schedules and consistent health management.

Understanding the presence of zoonotic bacteria in the gut microbiota of cats is critical for public health [38]. In this study, the results showed that the gut microbiota of stray cats was significantly enriched with a genus of opportunistic *Escherichia-Shigella* compared to domestic cats. Similar findings also were reported in another study conducted in Seoul, which revealed a 56% detection rate of *Escherichia-Shigella* in stray cats, markedly higher than the 12% observed in domestic cats [5]. Species of this genus not only cause gut infections in humans [39] but also show enrichment in cats with compromised immune function or organ dysfunction [40]. Therefore, the potential pathogenic bacteria of this genus may not only affect stray cats’ health but also spread to other animals or humans through them, which should be given due attention in the management of stray cats. In addition, the relative abundance of *Parasutterella* was also enriched in the stray group. As a genus of Gram-negative bacteria belonging to the Proteobacteria phylum, *Parasutterella* has been reported to have an increase in the relative abundance of this genus, which can lead to dysbiosis and a decrease in diversity of the gut microbiota [41]. Previous studies have shown that a decrease in the diversity of the gut microbiota is likely to increase the risk of opportunistic pathogen infections in the host [42,43]. Therefore, apart from living in an unclean environment for a long time, the significant increase in potential pathogenic bacteria in stray cats may also be related to the low diversity of their gut microbiota.

Moreover, domestic cats and stray cats exhibit significant differences in the predicted COG functions of their gut microbial community. Domestic cats’ gut microbiota primarily enriches functional genes related to homeostasis maintenance, cellular repair, and metabolic support, with abundant genes involved in cell wall biosynthesis, energy metabolism, and amino acid transport, as evidenced by a study on adult cats under dietary fiber interventions [44]. This indicates that under conditions of adequate nutrition and stable environments, domestic cats’ microbial communities mainly focus on maintaining physiological balance and immune homeostasis. In contrast, stray cats’ gut microbiota shows significant enrichment of functional genes associated with stress adaptation, pathogen defense, and secondary metabolite synthesis [33], which may reflect adaptive changes in their microbial communities when facing environmental stressors and pathogen pressures [33,45].

However, our current research still has several limitations. First, our research subjects were conducted within a single urban area and thus were unable to comprehensively reveal the characteristics of the gut microbiota and potential pathogen reservoirs of cats living in different regions and environments. Second, although the use of 16S rRNA molecular markers and high-throughput sequencing methods enables us to compare the composition and structure of the gut microbiota of domestic and stray cats, it is still difficult to conduct research at the species level. Further research integrating data from metagenomics, metabolomics, and resistanceomics will help elucidate the causes of microbial imbalance and pathogen proliferation. Applying molecular epidemiology techniques can also trace the transmission pathways of microbes among stray cats, domestic animals, and humans, thereby supporting a more integrated “One Health” approach.

## 5. Conclusions

This research revealed that stray cats exhibit a less diverse and unstable, more stress-adapted, and potentially pathogen-enriched microbial profile compared to domestic cats. Our results highlight the importance of monitoring the gut microbiota of urban stray cats. Regular surveillance helps in the early detection of emerging zoonotic threats and provides a basis for developing effective prevention and control strategies.

## Figures and Tables

**Table 1:** Sample information.

Sample ID	Sampling Date	Sex	Sampling Location
L1	17 March 2023	Female	Campus
L2	18 March 2023	Female	Campus
L3	15 March 2023	Female	Campus
L4	15 March 2023	Male	Residential Community
L5	28 March 2023	Male	Residential Community
L6	24 March 2023	Female	Campus
L7	2 March 2023	Female	Campus
L8	4 March 2023	Male	Residential Community
L9	7 March 2023	Female	Campus
L10	8 March 2023	Female	Animal Rescue Center
L11	22 March 2023	Male	Animal Rescue Center
L12	24 March 2023	Male	Animal Rescue Center
L13	26 March 2023	Male	Residential Community
L14	25 March 2023	Male	Residential Community
J1	19 December 2022	Female	Pet Store A
J2	25 December 2022	Male	Pet Store A
J3	27 December 2022	Female	Pet Store A
J4	9 December 2022	Male	Pet Store A
J5	20 December 2022	Male	Pet Store B
J6	23 December 2022	Female	Pet Store B
J7	14 December 2022	Female	Pet Store B
J8	16 December 2022	Male	Pet Store B
J9	10 December 2022	Female	Pet Store C
J10	21 December 2022	Female	Pet Store C
J11	26 December 2022	Male	Pet Store C

L: stray cat; J: domestic cat.

**Table 2:** Results of high-throughput sequencing of bacterial communities in different groups.

Sample Grouping	Number of Quality Filters	Number of ASVs
Stray cats (*n* = 14)	336,476	1593
Domestic cats (*n* = 11)	264,374	1310

## Data Availability

The raw reads were deposited into the NCBI Sequence Read Archive (SRA) database (accession number PRJNA1356635).
